# Fullerene Gallium Phosphonate Shows Antimycobacterial Effect Against *Mycobacterium avium*

**DOI:** 10.3390/ijms26209998

**Published:** 2025-10-14

**Authors:** Sonyeol Yoon, Kayvan Sasaninia, Iffat Hasnin Era, Sanya Dhama, Aishvaryaa Shree Mohan, Ami Patel, Lannhi Nguyen, Arshavir Karapetyan, Cristian Sy, Nickolas Yedgarian, Nezam Newman, Xiaoning Bi, Michel Baudry, Peter R. Yang, Vishwanath Venketaraman

**Affiliations:** 1 College of Osteopathic Medicine of the Pacific, Western University of Health Sciences, Pomona, CA 91766, USAnickolas.yedgarian@westernu.edu (N.Y.);; 2 College of Health Sciences, Western University of Health Sciences, Pomona, CA 91766, USA; nezam.newman@westernu.edu; 3 College of Dental Medicine, Western University of Health Sciences, Pomona, CA 91766, USA; 4 Sinapu LLC, 6 Technology Dr., Irvine, CA 92618, USA

**Keywords:** Fullerene Gallium Phosphonate, fullerene, *Mycobacterium avium*, oxidative stress

## Abstract

*Mycobacterium avium* complex (MAC) infections present significant therapeutic challenges due to their inherent antibiotic resistance, demanding innovative treatment approaches. This study investigated the antimicrobial and antioxidant potential of a novel compound, Fullerene Gallium Phosphonate (FGP), and compared its effects against a previously tested similar compound, Fullerene Disodium Phosphonate (FDSP). Results of experiments using MAC cultures and infected THP-1 macrophages treated with varying FGP and FDSP concentrations (1, 10, 100 µg/mL) revealed that FGP demonstrated greater efficacy than FDSP in reducing *M. avium* colony-forming units (CFU), achieving a nearly 3-fold reduction by day 8, compared to a 2-fold decrease with FDSP. In infected macrophages, FGP significantly decreased bacterial load at 1 and 10 µg/mL (*p* < 0.01). FGP also lowered oxidative stress, reflected by a significant reduction in malondialdehyde (MDA) levels on day 4 (*p* < 0.05) and decreased IL-6 (2-fold) and TNF-α levels (3-fold) by day 8, indicating both antimicrobial and anti-inflammatory effects. However, FGP paradoxically increased MAC burden at its highest concentration and showed no significant difference in efficacy of different concentrations. These findings suggest that FGP may serve as a promising candidate for antimycobacterial therapy with dual antibacterial and antioxidant effects. Further research is crucial to fully elucidate its mechanism of action and find the optimal therapeutic window.

## 1. Introduction

Nontuberculous Mycobacterial (NTM) infections are increasing in prevalence worldwide with over 190 NTM species identified in humans [1]. Of the identified species, *Mycobacterium avium complex* (MAC) infections are one of the most common causes of pulmonary NTM [2]. MAC is found ubiquitously in the environment, with reservoirs particularly in water sources and in the soil [2]. Immunocompromised individuals, in addition to those with chronic respiratory diseases, are at increased risk of acquiring MAC infections [3]. MAC infections are treated with a 12-month-long antibiotic regimen consisting of a combination therapy of rifampicin, azithromycin, clarithromycin, and/or ethambutol [4]. The treatment duration, along with toxicity and emerging antibiotic resistance in mycobacterial strains, presents challenges in the management of MAC infections and prompts a need for novel drug and therapeutic treatment modalities.

Buckminsterfullerene is a stable spherical icosahedral compound composed of 60 carbon atoms. The structure of buckminsterfullerene consists of a delocalized π double-bond system and sp2-hybridized carbons contributing to the structure’s strong electron-affinity towards multiple functional groups, offering promising prospects for their use in medicine [5]. Since its development, various pharmacological studies on buckminsterfullerene and its derivatives have been conducted for their antimicrobial activities. Fullerene compounds demonstrated antiviral, antifungal, and antibacterial effects, including inhibiting viral replication, bacterial cell membrane disruption, and bacterial metabolism inhibition [6,7,8]. Various reports also suggest that the efficacy of fullerenes as an antimicrobial agent can be attributed to high reactivity originating from the high surface-to-volume ratio and its cell membrane penetrance due to hydrophobic property [6,9]. Additionally, modulating the charge of the fullerene compounds has been demonstrated to increase their efficacy, with cationic derivatives having the highest antimicrobial effect compared to neutral and anionic derivatives [10].

Fullerene Gallium Phosphonate (FGP) is a novel buckminsterfullerene derivative combining buckminsterfullerene with gallium phosphonate functional groups. Fullerene and gallium have demonstrated broad-spectrum antimicrobial activity; however, there remains limited insight on the efficacy of fullerene phosphonate complexes specifically incorporating gallium. Given the increasing need for alternative antimicrobial strategies, FGP represents a promising candidate for further study, particularly for its potential applications against mycobacterial infections. Fullerene disodium phosphonate (FDSP) is a closely related derivative of fullerene that has the phosphonate group stabilized by sodium ions rather than gallium metal. FDSP has been greatly described in patent and formulation descriptions, though there is little known about its antimicrobial applications in peer-reviewed scientific literature. Because of the gap in research, we used FDSP for result comparisons to determine whether the introduction of gallium provides added antimicrobial benefits. Currently, there is limited data in peer-reviewed literature on the cytotoxicity of FGP and FDSP in mammalian cells. Therefore, we have summarized below the most relevant information on cytotoxicity evidence from fullerene and gallium systems. In this study, we aimed to assess the direct effects of FGP against *M. avium* in addition to its ability to clear intracellular infection. Furthermore, we also assessed the capacity for FGP to mitigate host cell oxidative stress and modulate the host immune response.

## 2. Results

### 2.1. Direct Effects of FGP and FDSP on M. avium

**Figure 1:**
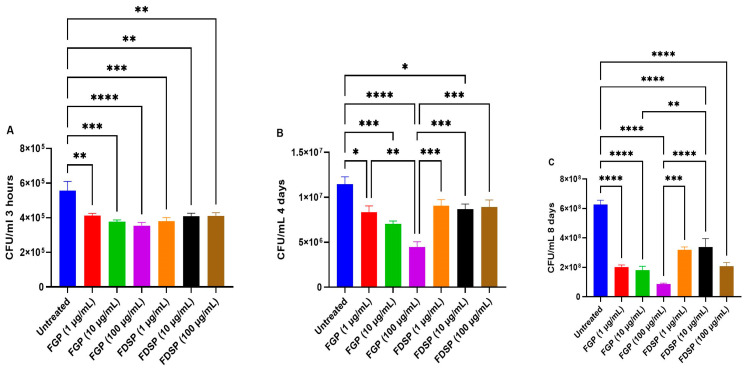
CFU counts of *M. avium* cultures treated with FGP, FDSP, or control. (**A**) CFU/mL of *M. avium* culture treated with 1, 10, and 100 μg/mL of FGP and FDSP at 3 h post-treatment. (**B**) CFU/mL of *M. avium* culture treated with 1, 10, and 100 μg/mL of FGP and FDSP at 4 days post-treatment. (**C**) CFU/mL of *M. avium* treated with 1, 10, and 100 μg/mL of FGP and FDSP at 8 days post-treatment. GraphPad Prism Software (Version 10.5.0) was utilized for analysis. Statistical analysis was performed using ANOVA. Asterisks (*) indicate a comparison between each group. *p*-values are indicated at the top of each graph, and <0.05 (*), <0.01 (**), <0.001 (***), <0.0001 (****).

Colony-forming unit (CFU) counts of *M. avium* cultures treated with FGP, FDSP, or control: A significant reduction in CFU count from the control value was observed 3 h post-infection with all concentrations (1, 10, and 100 μg/mL) of FGP or FDSP (Figure 1A). At 4 days post-infection, CFU counts of *M. avium* were significantly lower with all concentrations of FGP and FDSP (Figure 1B). We continued to observe a significantly reduced *M. avium* growth 8 days post-infection with a 3-fold reduction in CFU count in cultures treated with FGP and a nearly 2-fold reduction after treatment with FDSP (Figure 1C).

### 2.2. Efficacy of FGP and FDSP on Infected Host Cells

**Figure 2:**
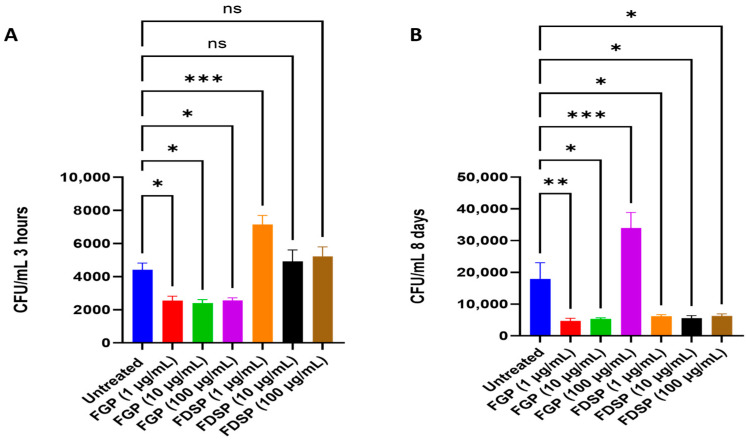
CFU counts of *M. avium* infected THP-1 cells treated with FGP, FDSP, or control. (**A**) CFU/mL of *M. avium* infected THP-1 cells treated with 1, 10, and 100 μg/mL of FGP and FDSP at 3 h post-treatment. (**B**) CFU/mL of *M. avium* infected THP-1 cells treated with 1, 10, and 100 μg/mL of FGP and FDSP at 8 days post-treatment. GraphPad Prism Software (Version 10.5.0) was utilized for analysis. Statistical analysis was performed using ANOVA. *p*-values are indicated at the top of each graph, and <0.05 (*), <0.01 (**), <0.001 (***). Nonsignificant *p*-values are indicated as ns.

CFU counts of *M. avium* infected THP-1 cells treated with FGP, FDSP, or control: A significant reduction in CFU count was observed 3 h post-infection with FGP treatment, while there was a significant increase in CFU count with 1 μg/mL of FDSP treatment (Figure 2A). At 8 days post-infection, *M. avium* CFU counts were significantly lowered with 1 and 10 μg/mL FGP treatments and all FDSP treatments, but they were increased with 100 µg/mL FGP (Figure 2B).

### 2.3. IL-6 Levels in the Supernatants of M. avium Infected THP-1 Cells with FGP/FDSP Treatment

**Figure 3:**
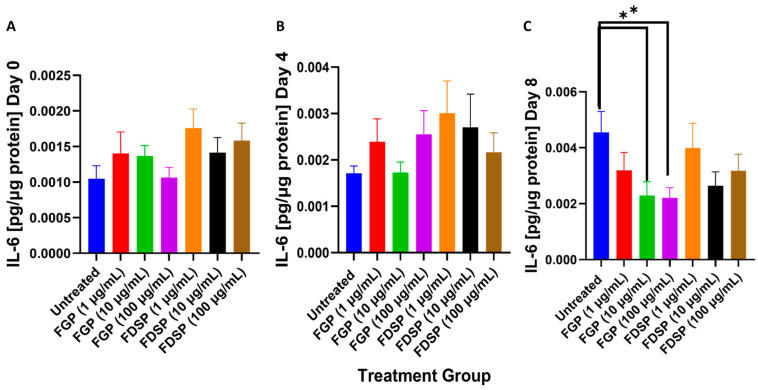
IL-6 levels in the supernatants of *M. avium* infected THP-1 cells with FGP/FDSP treatment or no treatment (untreated). (**A**) IL-6 levels of untreated and FGP/FDSP treated *M. avium* infected THP-1 macrophages 3 h (day 0) post-infection. (**B**). IL-6 levels of untreated and FGP/FDSP treated *M. avium* infected THP-1 macrophages 4 days post-infection (**C**) IL-6 levels of untreated and FGP/FDSP treated *M. avium* infected THP-1 macrophages 8 days post-infection. GraphPad Prism Software (Version 10.5.0) was utilized for analysis. Statistical analysis was performed using ANOVA. *p*-values are indicated at the top of each graph, and <0.05 (*).

Levels of IL-6 did not significantly differ between treatment groups 3 h post-infection nor 4 days post-infection (Figure 3A,B). At 8 days post-infection, a significant 2-fold decrease in IL-6 levels was observed with the 10 and 100 μg/mL FGP treatments (Figure 3C).

### 2.4. TNF-a Levels in the Supernatants of M. avium-Infected THP-1 Cells and Treated with FGP or FDSP

**Figure 4:**
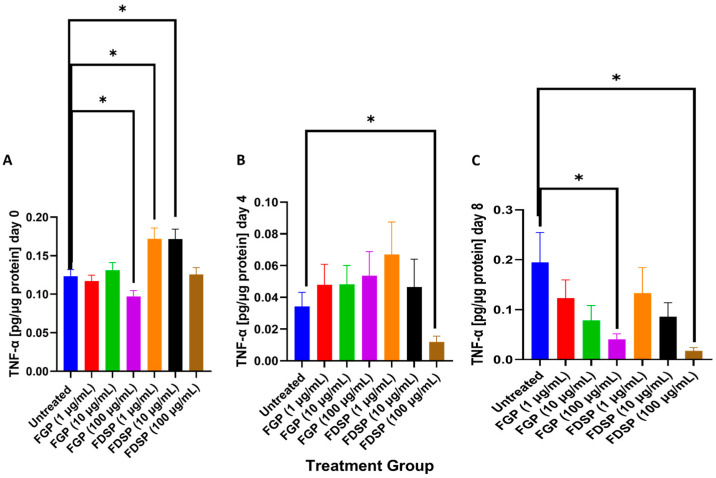
TNF-α levels in the supernatants of *M. avium*-infected and FGP/FDSP-treated THP-1 cells. (**A**) TNF-α levels of untreated and FGP/FDSP treated *M. avium*-infected THP-1 macrophages 3 h (day 0) post-infection. (**B**). TNF-α levels of untreated and FGP/FDSP treated *M. avium*-infected THP-1 macrophages 4 days post-infection (**C**) TNF-α levels of untreated and FGP/FDSP treated *M. avium*-infected THP-1 macrophages 8 days post-infection. GraphPad Prism Software (Version 10.5.0) was utilized for analysis. Statistical analysis was performed using ANOVA. *p*-values are indicated at the top of each graph, and <0.05 (*).

At 3 h post-infection, there were significant reductions in levels of TNF-α following treatment with 100 μg/mL FGP and 1 and 10 μg/mL FDSP (Figure 4A). Data from termination 4 days post-infection indicated a 3-fold significant decrease of TNF-α in the 100 μg/mL FDSP treatment (Figure 4B). The greatest significant reduction in TNF-α levels occurred in *M. avium*-infected macrophages treated with 100 μg/mL of FGP or FDSP 8 days post-infection (Figure 4C).

### 2.5. MDA Levels in M. avium-Infected THP-1 Cells Without Treatment (Control) or with FGP or FDSP Treatment

**Figure 5:**
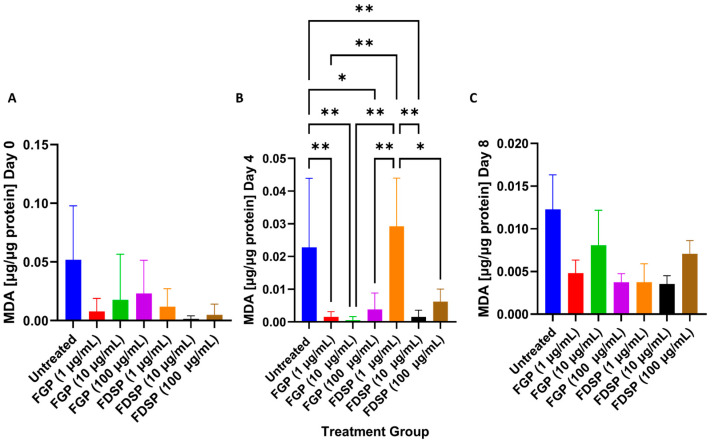
MDA levels in *M. avium-infected* THP-1 cells without treatment (control), FGP treatment, or FDSP treatment. (**A**) MDA levels in *M. avium* infected THP-1 cells with 1, 10, and 100 μg/mL of FGP and 1, 10, and 100 μg/mL of FDSP, respectively, at 3 h post-treatment; (**B**) MDA levels in *M. avium* infected THP-1 cells with 1, 10, and 100 μg/mL of FGP and 1, 10, and 100 μg/mL of FDSP, respectively, at 4 days post-treatment; (**C**) MDA levels in *M. avium* infected THP-1 cells with 1, 10, and 100 μg/mL of FGP and 1, 10, and 100 μg/mL of FDSP, respectively, at 8 days post-treatment. GraphPad Prism Software (Version 10.5.0) was utilized for analysis. Statistical analysis was performed using ANOVA. *p*-values are indicated at the top of each graph, and <0.05 (*), <0.01 (**).

At 4 days post-infection, there were significant reductions in MDA levels after 1, 10, and 100 μg/mL FGP treatment and 10 μg/mL FDSP (Figure 5B). Treatments 3 h and 8 days post-infection did not show significant differences (Figure 5A,C)

## 3. Discussion 

Results from previous studies indicated that FGP functions as a free radical scavenger, reducing oxidative stress, weakening bacterial defenses, and increasing susceptibility to other antimicrobial agents [11,12,13,14]. Additionally, FGP appears to inhibit certain bacterial enzymes, further impairing microbial survival [15]. Given that metal-based nanoparticles have been reported to enhance the efficacy of conventional antibiotics [10], investigating the potential synergy between fullerene derivatives and antibiotics in antimicrobial applications is of particular interest. This study explored its specific effects on mycobacteria, particularly *M. avium*, and compared them to those of another fullerene compounds previously tested, FDSP.

Specifically, the study sought to (1) compare the efficacy of FGP and FDSP against *M. avium*, (2) determine whether FGP exerts a direct antimycobacterial effect, and (3) validate gallium as a core component of FGP by comparing its performance to FDSP. To test these hypotheses, in vitro experiments were conducted using *M. avium*-infected THP-1 macrophages, with bacterial load measured through CFU counts and immune and oxidative stress response modulation assessed via cytokine assays, IL-6, and TNF-α, and MDA, respectively. We predicted that while both FGP and FDSP would exhibit antimycobacterial activity, the presence of gallium would confer greater efficacy. This study was designed as a proof-of-concept experimentation of FGP rather than comparison with currently available antimycobacterial drugs, so control antibiotics were not included in the experimental design.

Gallium (III) (Ga^3+^) is a post-transition metal that has been under investigation for its antimicrobial effect especially against multidrug-resistant infections [10]. At room temperature, gallium remains as a liquid, which allows for its decomposition into nanoparticles by ultrasound and subsequently could act as drug carriers [16]. This use of metal-based nanoparticles convinced many researchers that gallium could reduce microbial resistance to antibiotics, thereby supporting its therapeutic use [17].

The current understanding of gallium antimicrobial effect is that it acts as an iron mimic with its structural and chemical similarity. Unlike iron, gallium is an inert metal that does not undergo reduction–oxidation reactions under physiological conditions. Therefore, it is thought to interact with microbial proteins, and interfere with their vital metabolic functions, thereby inhibiting microbial proliferation [18]. This likely occurs after gallium is internalized by the microbe and interacts with catalase and superoxide dismutase to inhibit the bacterial antioxidant system. Subsequently, the increase in susceptibility to oxidation makes the microbes vulnerable to reactive oxygen species [19]. In support of this, physical destruction of bacterial biofilms by changing reaction conditions and production of reactive oxygen species have been observed in studies with gallium [19].

The antimicrobial potential of gallium and its derivatives was reported decades ago, with early studies demonstrating that gallium tartrate could eradicate *Treponema pallidum* and *Trypanosoma evansi* in animals [20]. Investigations persevered throughout time and various reports have shown that gallium may be effective against a variety of bacterial species, including those known for multidrug resistance, such as *Mycobacterium tuberculosis*, *Klebsiella pneumoniae*, and *Pseudomonas aeruginosa* [21,22,23]. Additionally, a study by Chitambar et al. showed that gallium nitrate and transferrin-gallium block *Mycobacterium avium*’s iron-dependent growth within human macrophages [24]. However, Gallium (III) ions have extremely low bioavailability in the body due to their hydrolyzable property under physiologic conditions and studies are currently ongoing to optimize gallium with various functional groups that would maximize both bactericidal effects and bioavailability [18,25].

In preliminary experiments, FGP demonstrated antiviral activity against SARS-CoV-2, although its precise mechanism of action remains to be fully elucidated [26]. One proposed mechanism involves reversible hopping between gallium and sodium ions via π–bond interactions, a process that imparts nano-surfactant properties to the compound [26]. This allows FGP to interact with viral proteins, which are typically stabilized by van der Waals forces, potentially disrupting viral integrity [26]. Furthermore, the displacement of gallium phosphonate by sodium ions is believed to facilitate FGP’s attachment to negatively charged bacterial membranes, enhancing its ability to penetrate and disrupt microbial cells [26].

In this study, we first assessed the direct effect of FGP on *M. avium* in culture using a time-kill assay. The methodology enables exploration of the dose–response relationship and time-dependent effects of the FGP treatments. Testing differing concentrations can lead to identification of an optimal concentration range to achieve antimycobacterial effect via synergistic effects [27]. This assay allowed us to observe the immediate impact of a compound (3 h post-treatment), in addition to its efficacy after subsequent treatments (4 days post-infection with two treatments) and sustained infection (8 days, three treatments). We observed a dose-dependent response to increasing concentrations of FGP at all time points, demonstrating sustained antimycobacterial activity (Figure 1A–C). In the absence of gallium (FDSP), a significant decrease in *M. avium* growth was also observed compared to the untreated control; however, the effect size was diminished compared to FGP. This finding supports our initial hypothesis that the inclusion of gallium confers a more potent fullerene formulation when interacting with *M. avium*.

We then assessed the effects of FGP in an infected *M. avium* THP cell model to ascertain whether FGP can exert its antimycobacterial effect intracellularly. During an infection, *M. avium* comes into contact with a resident macrophage, where it is phagocytosed. However, *M. avium* resists phagocytic degradation and persists intracellularly where it continues to replicate [23]. FGP treatment significantly reduced the *M. avium* burden at all concentrations 3 h post-infection compared to the untreated control and FDSP, which is consistent with our initial finding (Figure 2A). However, no significant difference between concentrations of FGP was observed. This finding could indicate that FGP reaches its maximal effect at the lowest concentration. At 8 days post-infection, both FGP and FDSP elicited similar antimycobacterial effects (Figure 2B). Paradoxically, FGP treatment at 100 ug/mL resulted in significantly higher *M. avium* burden compared to all other treatment categories. The reason for this finding is unclear, although such inverted dose–response curves are quite frequent in pharmacology. It is possible that high doses of FGP may have activated mycobacterial stress responses, enabling survival from high concentrations of antibiotics, though further studies are needed [28].

*M. avium* infection is known to generate oxidative stress [29]. Host immune cells, such as macrophages, utilize reactive oxygen species (ROS) or reactive nitrogen species (RNS) to eliminate pathogens; however, excess production of ROS/RNS can have host-detrimental effects. We determined whether FGP possesses antioxidant capacity by measuring MDA levels. MDA is a byproduct of lipid peroxidation due to ROS damage and serves as a measurement of oxidative stress. FGP treatment significantly reduces MDA levels 4 days post-infection compared to untreated and FDSP treatment groups, indicating that gallium enhances the capacity for FGP to serve as an antioxidant (Figure 5B).

To corroborate this finding, we also measured cytokine levels generated in response to oxidative stress. Cellular damage resulting from reactive oxygen/nitrogen species (ROS/RNS) activates intracellular signaling pathways involving nuclear factor kappa B (NF-kB) and mitogen-activated protein kinase (MAPK) promoting transcription and expression of IL-6 [30,31,32]. IL-6 is a cytokine that activates the expression of acute phase proteins that scavenge free radical species [31]. Furthermore, IL-6 activates signal transducer and activator of transcription 3 (STAT3) which in turn increases the expression of several antioxidant defenses [31,32]. We observed that FGP treatment significantly reduced IL-6 levels compared to untreated controls indicating that the gallium component in FGP plays a role in reducing the host cell response to oxidative stress by decreasing ROS (Figure 3A–C).

TNF-α is another cytokine under the activation of NF-kB and a classical marker for M1 macrophage activation [33]. FGP treatment demonstrated a dose-dependent decrease in TNF-α levels 8 days post-infection; FDSP treatment also showed a similar trend (Figure 4C). This finding may indicate that fullerene compounds can modulate the macrophage response independently of gallium.

There are limitations to this study. This study only illustrates the downstream effects of FGP on *M. avium* survival and modulation of the host cell response to *M. avium* infection. Further studies are needed to evaluate the mechanism of action of FGP. Furthermore, cytotoxicity assays are required to assess the therapeutic viability of fFGP in living systems. Additionally, there are still ongoing investigations of appropriate functionalization of fullerene compounds and their derivatives, as their low solubility in polar solvents poses barriers to their potential applications [34].

## 4. Materials and Methods

### 4.1. Materials

**Figure 6:**
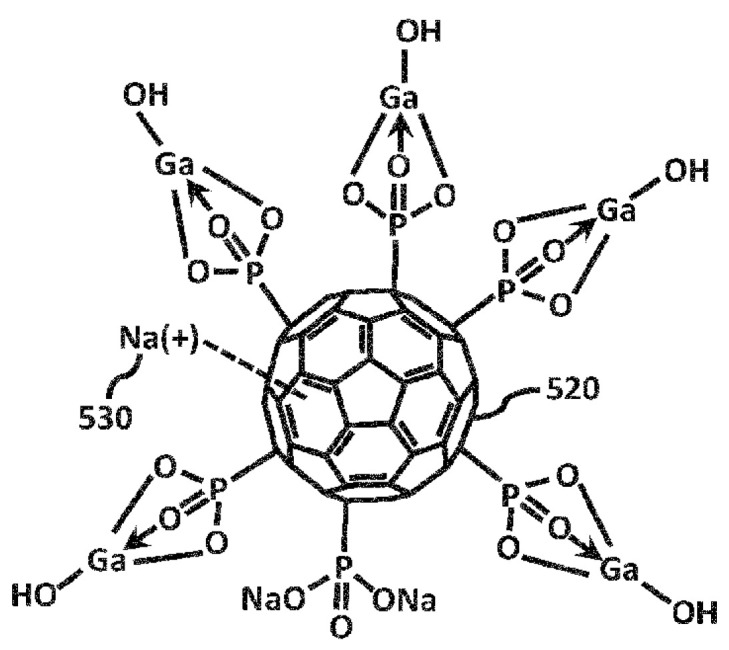
Chemical structure of Fullerene Gallium Phosphonate (FGP). Structure obtained and formatted for clarity from European Patent Application EP4292656A1 [35].

**Figure 7:**
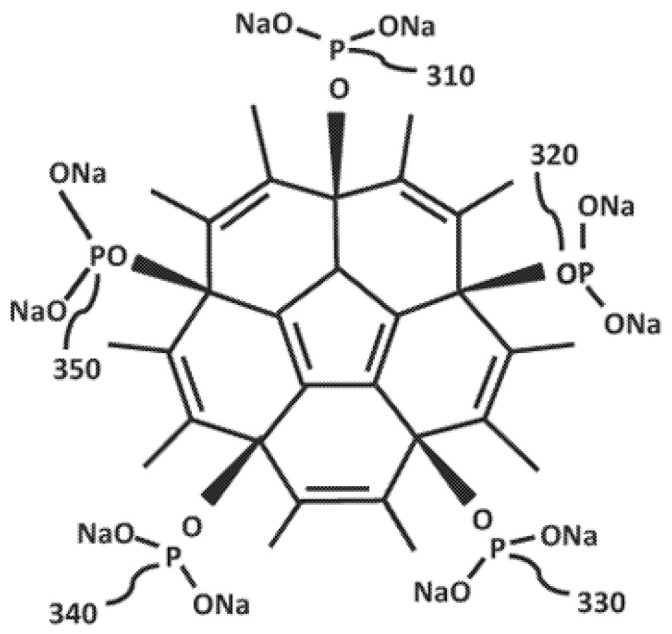
Chemical structure of Fullerene Disodium Phosphonate (FDSP). Structure obtained and formatted for clarity from International Patent Application WO2022159332A1 [36].

Fullerene Gallium Phosphonate (FGP) and Fullerene disodium phosphonate (FDSP) were provided by Dr. Peter Butzloff (Sinapu LLC., Irvine, CA, USA). The chemical structures of FGP and FDSP are shown in Figure 6 and Figure 7 respectively:

### 4.2. Bacterial Processing and Preparation

All experiments utilized a laboratory strain of *M. avium* derived from ATCC 25291™, obtained via KWIKSTIK™ (Microbiologics, St. Cloud, MN, USA). The bacteria were cultured in 7H9 medium (Hi Media, Santa Maria, CA, USA), supplemented with albumin dextrose complex (ADC) (Gemini, New York City, NY, USA), and incubated at 37 °C in a 5% CO_2_ until reaching the logarithmic growth, indicated by an optical density (OD_600_) of 0.5 to 0.8. *M. avium* cultures were then harvested and processed to obtain a single-cell suspension by disrupting bacterial clumps. The bacteria were centrifuged (≈2020× *g*, 25 °C, 12 min) and rinsed with a 1x phosphate-buffered saline (PBS) (Sigma, St. Louis, MO, USA) before being vortexed with 3 mm sterile glass beads at 3 min intervals. To further separate bacterial clumps, the vortexed bacteria were filtered through a 5 µm filter. Processed *M. avium* was serially diluted, plated on 7H11 agar (Hi Media, Santa Maria, CA, USA), and incubated at 37 °C to determine bacterial count. Aliquots of the prepared stock were dispensed into individual tubes and stored in a cryogenic box in −80 °C until use. All procedures were performed aseptically within a Class II biosafety cabinet.

### 4.3. THP-1 Cell Differentiation, Infection, and Antibiotic Treatment

THP-1 cells from ATCC were cultured in RPMI-1640 medium (Sigma, St. Louis, MO, USA), and maintained in a 37 °C incubator with 5% CO_2_. The cells were then harvested for subsequent experiments. Prior to the experiments, THP-1 cells were counted using a hemocytometer and trypan blue stain. Each well of a 96-well tissue culture plate was coated with a poly-L-lysine solution for 1 h. The harvested THP-1 cells were then treated with a 10 ng/mL solution of phorbol 12-myristate 13-acetate (PMA). The PMA-treated THP-1 cells were then added to each well of the 96-well tissue culture plate (Corning, Corning, NY, USA) at 2 × 10^5^ per well. The plate was then placed in a 37 °C incubator with 5% CO_2_ overnight to facilitate the differentiation of cells into macrophages before day 0. The next day, each well was examined under a microscope to confirm the formation of a differentiated monolayer of cells.

The supernatant was then removed from each well and replaced with RPMI with 10% fetal bovine serum (FBS) infected with *M. avium* at a 1:1 concentration of *M. avium* to THP-1 cells (2 × 10^5^ *M. avium*: 2 × 10^5^ THP-1 cells). The 96-well plate was then placed in a 37 °C incubator with 5% CO_2_ for 1 h. Following the incubation period, the supernatant was discarded and phagocytosed bacteria were removed by washing with a 1×PBS solution three times. Fresh RPMI with 10% FBS was then added to each well. Various treatments were then administered to their corresponding wells, including FGP and FDSP at various concentrations. Compounds were added in nanopure water and sterilized through heat sterilization. Compounds were sonicated to ensure homogenization prior to treatment. PBS was used as a control for untreated cells.

Each treatment group was cultured in triplicate in the 96-well plate. Treatments were added immediately after *M. avium* infection, 3 days post-infection, and 6 days post-infection. After adding the treatments to their respective wells, the plate was incubated at 37 °C incubator with 5% CO_2_. Sections of the 96-well plate were terminated 3 h (Day 0), 4 days, and 8 days post-infection. To terminate each well, the supernatant was collected for biomarker analysis, and ice-cold nano-pure water was added in its place. Slight friction was delivered to release the cells and the entire contents of each well were removed. The contents of each well were spread onto MiddleBrook 7H11 Agar Medium in duplicate and placed in a 37 °C incubator without CO_2_ for 11 days. After incubation, *M. avium* colonies were counted.

### 4.4. Cytokine Measurement

Interleukin-6 (IL-6) and Tumor Necrosis Factor-α (TNF-α) levels were assessed in the supernatant of *M. avium*-infected macrophages treated with 1, 10, and 100 μg/mL of FGP and FDSP and a control across three treatment and termination timepoints using enzyme-linked immunosorbent assay (ELISA) kits obtained from ThermoFisher Scientific (ThermoFisher Scientific, Waltham, MA, USA) using the manufacturer’s instructions [37,38]. Specifically, the following ELISA kits were used: IL-6 Mouse Uncoated ELISA Kit (Cat# 88-7064-88), and TNF-α Mouse Uncoated ELISA Kit (Cat# 88-7324-88). IL-6 and TNF-α were measured in culture supernatants using the mouse uncoated ELISA kits (ThermoFisher Scientific; Cat# 88-7064-88 and 88-7324-88) following the manufacturer’s protocol. Standards and samples were added to the pre-coated plates, incubated with detection and capture antibodies, followed by streptavidin and substrate solution. The absorbance was read at 450 nm using a microplate reader. All measurements were normalized to the total protein levels of the samples and reported as picograms of cytokine per microgram of protein.

### 4.5. Malondialdehyde Measurement

Malondialdehyde (MDA) levels from treated THP-1 cells were measured spectrophotometrically using a Cayman Chemicals Thiobarbituric Acid Reactive Substances (TBARS) Assay Kit (catalog #10009055), following the manufacturer’s protocols (Cayman Chemicals, Ann Arbor, MI, USA) [39]. Samples were mixed in thiobarbituric acid solution, heated to promote adduct formation, cooled, and had the absorbance read at 532 nm using a microplate reader. MDA measurements were normalized to total sample protein levels and reported as micromoles of MDA per microgram protein.

## 5. Conclusions

The study demonstrated that FGP exhibits antimycobacterial activity against *Mycobacterium avium*, both extracellularly and within infected macrophages. The greater efficacy of FGP compared to its counterpart, FDSP, supports the hypothesis that gallium could be considered as a core antimicrobial component in fullerene derivatives. FGP also exhibited antioxidant properties as shown by reduced MDA and IL-6 levels, which suggests its dual role in both pathogen clearance and host cell protection. Given that antimycobacterial activity did not differ significantly with increasing concentrations, our results indicate that FGP could exert its maximal effect at lower concentrations with no added benefit at higher doses. Furthermore, the paradoxical increase in *M. avium* burden in its highest tested dose at 100 µg/mL raises the possibility that excessive FGP concentrations might induce bacterial stress to enhance their survival. Further investigations are needed for clarifications of these observations and to determine the mechanism and the optimal therapeutic window for FGP efficacy. Because the aim of the study was to test the effects of FGP against *M. avium*, a head-to-head comparison against current frontline antibiotics was not performed, although that may be a logical next step in order to better test the efficacy of FGP against currently available drugs. Additionally, further studies will be needed to evaluate pharmacodynamics, cytotoxicity in uninfected cells, and in vivo efficacy to determine whether the compound may contribute to simpler or shorter regimens.

## Data Availability

The data supporting reported results can be obtained from the corresponding author (V.V.) upon formal requisition.
